# Complete chloroplast genome sequencing of five *Salix* species and its application in the phylogeny and taxonomy of the genus

**DOI:** 10.1080/23802359.2021.1950055

**Published:** 2021-07-15

**Authors:** Jie Zhou, Zhongyi Jiao, Jiahui Guo, Bao song Wang, Jiwei Zheng

**Affiliations:** a National Willow Engineering Technology Research Center, Jiangsu Academy of Forestry, Nanjing, China; b College of Biology and the Environment, Nanjing Forestry University, Nanjing, China

**Keywords:** *Salix*, *Salix argyracea*, *Salix dasyclados*, *Salix eriocephala*, *Salix integra* ‘Hakuro Nishiki’, *Salix suchowensis*, chloroplast genome, phylogeny, taxonomy

## Abstract

In this study, whole chloroplast genomes of five *Salix* species (*S. argyracea*, *S. dasyclados*, *S. eriocephala*, *S. integra* ‘Hakuro Nishiki’, and *S. suchowensis*) were sequenced. These chloroplast genomes were 155 ,605, 155, 763, 155, 552, 155, 538, and 155 ,550 bp in length, harboring 131 genes (77 unigenes), 37 tRNA genes, 8 rRNA genes, and 86 mRNA genes, respectively. The genes *ycf1*, *psaI*, *ycf2-2*, *rpoC2*, *rpl22*, *atpF*, and *ndhF* were under positive selection among the 21 *Salix* species. *psaI, ycf2-2*, atpF, and *ycf1-2* were under positive selection between the tree willow and shrub willow, and *rpoC2*, *rpl22*, and *ycf1-2* were positively selected among the shrub genomes. The gene *rps7* was most variable among the genomes. Phylogenetic analysis of 21 *Salix* species and *Chosenia arbutifolia* provide evidence that the cp genome data partially support the relationship with traditional taxonomic concepts in the Flora of China. This chloroplast genome elucidates *Salix* taxonomy and provides evidence for evolutionary research.

## Introduction

Chloroplast DNA (cpDNA) is maternally inherited, thus providing essential information for molecular markers, breeding of new varieties, and plant phylogeny (Cui et al. 2019; Njuguna et al. 2019). The willow genus (*Salix* spp.) is composed of 350–520 species that are distributed worldwide. In the ‘Flora of China’, the species distributed in China are classified into 37 groups (Wang and Shi 2019). The five species sequenced here (*S. argyracea*, *S. dasyclados*, *S. eriocephala*, *S. integra* ‘Hakuro Nishiki’, and *S. suchowensis)* are widely planted in Jiangsu Province and produce a large amount of biomass. *Salix eriocephala* was introduced from the United States for its high biomass yield and as a source of bioenergy. All these species absorb the heavy metal cadmium (Cd) in their roots and are the most promising candidates for phytoremediation among the willow species. In addition, the leaves and flowers have great ornamental value. *Salix integra* ‘Hakuro Nishiki’ is available from nurseries in shrub and tree form with vibrant white and pink leaves. *Salix argyracea*, *S. suchowensis*, and *S. dasyclados* are widely used in crafts for wickerwork and decorations. Thus, sequencing of the cpDNA and molecular marker mining will be effective methods to segregate willow germplasms and reveal phylogenetic relationships.

## Materials and methods

### Plant materials

The five *Salix* species were collected and deposited in the willow collection at Jiangsu Academy of Forestry (31.861947°N, 118.777145°E). The voucher specimens of *S. argyracea*, *S. dasyclados*, *S. eriocephala*, *S. integra* ‘Hakuro Nishiki’, and *S. suchowensis* were deposited at the herbarium of Jiangsu Academy of Forestry under the voucher numbers P102, P126, 87, P646, and P63, respectively. The email of the person who is in charge of the sample collection is zjwin718@126.com.

### cpDNA sequencing and *de novo* assembly

Fresh leaves were collected for DNA isolation and library construction, and the DNA samples were stored at Key Laboratory of Jiangsu Academy of Forestry, Nanjing, China. Genomic sequencing was performed using the Illumina Novaseq PE150 platform (San Diego, CA, USA). The raw data were sequenced and filtered using fastp (version 0.20.0, https://github.com/OpenGene/fastp) software to obtain clean data. Then *de novo* assembly was constructed using SPAdes v3.10.1 (http://cab.spbu.ru/software/spades/) for the complete pseudo genome.

### Chloroplast gene annotation, selective press analysis and phylogenetic analysis

The cpDNA coding sequence was annotated using GeSeq (https://chlorobox.mpimp-golm.mpg.de/geseq-app.html) and visually checked in Geneious v8.0.2 (Kearse et al. 2012). The rRNA and tRNA were predicted using HMMER v3.1b2 (http://hmmer.org/) and ARAGORN v1.2.38 (Laslett and Canback 2004). The sequences were aligned using MAFFT v7.427 (https://mafft.cbrc.jp/alignment/software/). The Ka/Ks value was calculated using KaKs_Calculator v2.0 (https://sourceforge.net/projects/kakscalculator2/). Vcftools was used to calculate the Pi (Nucleotide diversity) value of every gene. The phylogenetic tree was constructed in MrBayes v3.2.7 with the Markov chain Monte Carlo (MCMC) methods and 1000 bootstrap replicates.

## Results

### Characterization of chloroplast genomes in Salix

The complete chloroplast (cp) genomes of *S. argyracea, S. dasyclados, S. eriocephala, S. integra* ‘Hakuro Nishiki’, and *S. suchowensis* were 155, 605, 155 ,763, 155 ,552, 155, 538, and 155 ,550 bp in size, respectively. The GC content of the IR, LSC, and SSC regions was approximately 41%, 30%, and 34%, respectively. It encodes 131 genes (77 unigenes), 37 tRNA genes, 8 rRNA genes, and 86 mRNA genes. The genomes exhibited a typical quadripartite structure with the LSC region (84,414–84,588 bp), SSC region (16,214–16,275 bp), and IRs (27,384–27,479 bp). Fourteen genes (*ndhA*, *ndhB*, *petB*, *petD*, *atpF*, *rpl16*, *rpl2*, *rpoC1*, *trnA-UGC*, *trnG-GCC*, *trnI-GAU*, *trnK-UUU*, *trnL-UAA*, and *trnV-UAC*) had one intron, and three genes (*rps12*, *clpP*, and *ycf3*) had two introns (Table 1).

**Table 1.** Annotated genes of the chloroplast genome of the five *Salix* species.

Category	Gene group	Gene name
Photosynthesis	Subunits of photosystem I	*psaA*, *psaB*, *psaC*, *psaI*, *psaJ*
	Subunits of photosystem II	*psbA*, *psbB*, *psbC*, *psbD*, *psbE*, *psbF*, *psbH*, *psbI*, *psbJ*, *psbK*, *psbL*, *psbM*, *psbN*, *psbT*, *psbZ*
	Subunits of NADH dehydrogenase	ndhA*, ndhB*(2), *ndhC*, *ndhD*, *ndhE*, *ndhF*, *ndhG*, *ndhH*, *ndhI*, *ndhJ*, *ndhK*
	Subunits of cytochrome b/f complex	*petA*, petB*, petD*, *petG*, *petL*, *petN*
	Subunits of ATP synthase	*atpA*, *atpB*, *atpE*, atpF*, *atpH*, *atpI*
	Large subunit of rubisco	*rbcL*
	Subunits protochlorophyllide reductase	*–*
Self-replication	Proteins of large ribosomal subunit	*rpl14*, rpl16*, rpl2*(2), *rpl20*, *rpl22*, *rpl23*(2), *rpl33*, *rpl36*
	Proteins of small ribosomal subunit	*rps11*, rps12**(2), *rps14*, *rps15*, *rps18*, *rps19*(2), *rps2*, *rps3*, *rps4*, *rps7*(2), *rps8*
	Subunits of RNA polymerase	*rpoA*, *rpoB*, rpoC1*, *rpoC2*
	Ribosomal RNAs	*rrn16*(2), *rrn23*(2), *rrn4.5*(2), *rrn5*(2)
	Transfer RNAs	trnA-UGC*(2), *trnC-GCA*, *trnD-GUC*, *trnE-UUC*, *trnF-GAA*, trnG-GCC*, *trnG-UCC*, *trnH-GUG*, *trnI-CAU*(2), trnI-GAU*(2), trnK-UUU*, *trnL-CAA*(2), trnL-UAA*, *trnL-UAG*, *trnM-CAU*, *trnN-GUU*(2), *trnP-UGG*, *trnQ-UUG*, *trnR-ACG*(2), *trnR-UCU*, *trnS-GCU*, *trnS-GGA*, *trnS-UGA*, *trnT-GGU*, *trnT-UGU*, *trnV-GAC*(2), trnV-UAC*, *trnW-CCA*, *trnY-GUA*, *trnfM-CAU*
Other genes	Maturase	*matK*
	Protease	clpP**
	Envelope membrane protein	*cemA*
	Acetyl-CoA carboxylase	*accD*
	c-type cytochrome synthesis gene	*ccsA*
	Translation initiation factor	#*infA*
	Other	*–*
Genes of unknown function	Conserved hypothetical chloroplast Opening Reading Frame (ORF)	*ycf1*(2), *ycf15*(2), *ycf2*(2), ycf3**, *ycf4*

*: Genes with one intron; **: Genes with two introns; ^#^: Pseudogene; (2) after gene name: Number of copies of multi-copy genes.

### Positive selection genes

**Figure 1.**
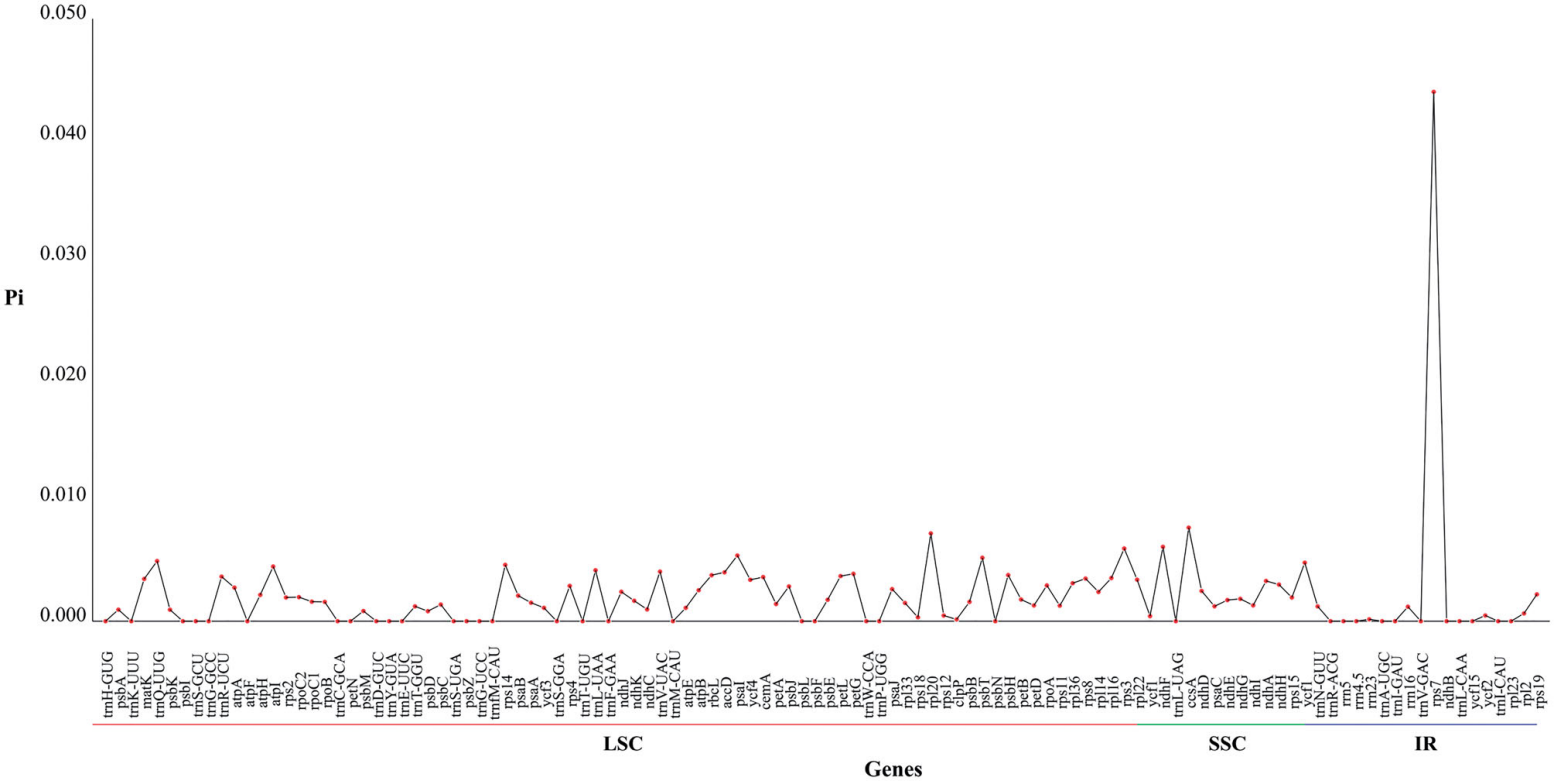
Nucleotide variability (Pi) values of 22 chloroplast genomes of *Salix* and *C. arbutifolia*.

The nonsynonymous substitution rate (Ka), synonymous substitution rate (Ks), and their ratio (Ka/Ks) are commonly used to calculate the direction of evolution and its selective strength in protein-coding genes. The genes *ycf1*, *psaI*, *ycf2-2*, *rpoC2*, *rpl22*, *atpF*, and *ndhF* were under positive selection in the 21 *Salix* species (Ka/Ks > 1) (Table 2). The gene *rps7*, located in the IR region, occupied the highest Pi value (Figure 1), indicating that the gene is the most variable among the 21 *Salix* genomes that could be used as potential molecular markers.

**Table 2.** Positive selection genes among the cp genomes.

Sequence	*Ka/Ks*	*p*-Value (Fisher)
*S. argyracea*_*psaI* vs *S. babylonica*_*psaI*	1.10156	0.317442
*S. argyracea*_*ycf2-2* vs *S. babylonica*_*ycf2-2*	1.11572	0
*S. argyracea*_*atpF* vs *S. paraplesia*_*atpF*	1.26289	0.621226
*S. argyracea*_*atpF* vs *S. tetrasperma*_*atpF*	1.26289	0.621226
*S. dasyclados*_*psaI* vs *S. babylonica*_*psaI*	1.10156	0.317442
*S. dasyclados*_*ycf2-2* vs *S. babylonica*_*ycf2-2*	1.17334	0
*S. dasyclados*_*atpF* vs *S. paraplesia*_*atpF*	1.26289	0.621226
*S. dasyclados*_*atpF* vs *S. tetrasperma*_*atpF*	1.26289	0.621226
*S. eriocephala*_*psaI* vs *S. babylonica*_*psaI*	1.10156	0.317442
*S. eriocephala*_*ycf2-2* vs *S. babylonica*_*ycf2-2*	1.11572	0
*S. eriocephala*_*atpF* vs *S. paraplesia*_*atpF*	1.26289	0.621226
*S. eriocephala*_*atpF* vs *S. tetrasperma*_*atpF*	1.26289	0.621226
*S. integra*_*psaI* vs *S. babylonica*_*psaI*	1.10156	0.317442
*S. integra* ‘Hakuro Nishiki’_*ycf2-2* vs *S. babylonica*_*ycf2-2*	1.11572	0
*S. integra* ‘Hakuro Nishiki’_*atpF* vs *S. paraplesia*_*atpF*	1.26289	0.621226
*S. integra* ‘Hakuro Nishiki’_*atpF* vs *S. tetrasperma*_*atpF*	1.26289	0.621226
*S. suchowensis*_*psaI* vs *S. babylonica*_*psaI*	1.10156	0.317442
*S. suchowensis*_*atpF* vs *S. paraplesia*_*atpF*	1.26289	0.621226
*S. suchowensis*_*ycf1-2* vs *C. arbutifolia*_*ycf1-2*	1.17113	0
*S. argyracea*_*ycf1-2* vs *C. arbutifolia*_*ycf1-2*	1.17113	0
*S. dasyclados*_*matK* vs *C. arbutifolia*_*matK*	1.28293	0.349676
*S. dasyclados*_*ycf1-2* vs *C. arbutifolia*_*ycf1-2*	1.17264	0
*S. eriocephala*_*ycf1-2* vs *C. arbutifolia*_*ycf1-2*	1.17113	0
*S. integra* ‘Hakuro Nishiki’_*ycf1-2* vs *C. arbutifolia*_*ycf1-2*	1.20107	0
*S. suchowensis*_*ycf1-2* vs *C. arbutifolia*_*ycf1-2*	1.17113	0
*S. argyracea*_*rpoC2* vs *S. dasyclados*_*rpoC2*	1.27437	0.34553
*S. argyracea*_*ycf1-2* vs *S. integra*_*ycf1-2*	1.35202	0.379283
*S. argyracea*_*rpl22* vs *S. interior*_*rpl22*	1.24719	0.353952
*S. dasyclados*_*rpoC2* vs *S. brachista*_*rpoC2*	1.27468	0.345621
*S. dasyclados*_*rpl20* vs *S. brachista*_*rpl20*	1.3916	0.787437
*S. dasyclados*_*rpoC2* vs *S. eriocephala*_*rpoC2*	1.27437	0.34553
*S. dasyclados*_*rpoC2* vs *S. gracilistyla*_*rpoC2*	1.27437	0.34553
*S. dasyclados*_*rpl22* vs *S. interior*_*rpl22*	1.24719	0.353952
*S. dasyclados*_*rpoC2* vs *S. minjiangensis*_*rpoC2*	1.27437	0.34553
*S. dasyclados*_*ndhF* vs *S. oreinoma*_*ndhF*	1.07235	0.288682
*S. dasyclados*_*rpoC2* vs *S. rehderiana*_*rpoC2*	1.27437	0.34553
*S. dasyclados*_*rpoC2* vs *S. suchowensis*_*rpoC2*	1.27437	0.34553
*S. dasyclados*_*rpoC2* vs *S. taoensis*_*rpoC2*	1.32528	0.365324
*S. eriocephala*_*rpl22* vs *S. interior*_*rpl22*	1.24719	0.353952
*S. integra* ‘Hakuro Nishiki’_*ycf1-2* vs *S. brachista*_*ycf1-2*	1.35049	0.378801
*S. integra* ‘Hakuro Nishiki’_*ycf1-2* vs *S. hypoleuca*_*ycf1-2*	1.12598	0.305479
*S. integra* ‘Hakuro Nishiki’_*ycf1-2* vs *S. koriyanagi*_*ycf1-2*	1.35202	0.379283
*S. integra* ‘Hakuro Nishiki’_*ndhF* vs *S. oreinoma*_*ndhF*	1.83448	0.846122
*S. integra* ‘Hakuro Nishiki’_*ycf1-2* vs *S. rorida*_*ycf1-2*	1.35185	0.379231
*S. integra* ‘Hakuro Nishiki’_*ycf1-2* vs *S. suchowensis*_*ycf1-2*	1.35202	0.379283
*S. suchowensis*_*rpl22* vs *S. interior*_*rpl22*	1.24719	0.353952

### Phylogenic analysis

**Figure 2.**
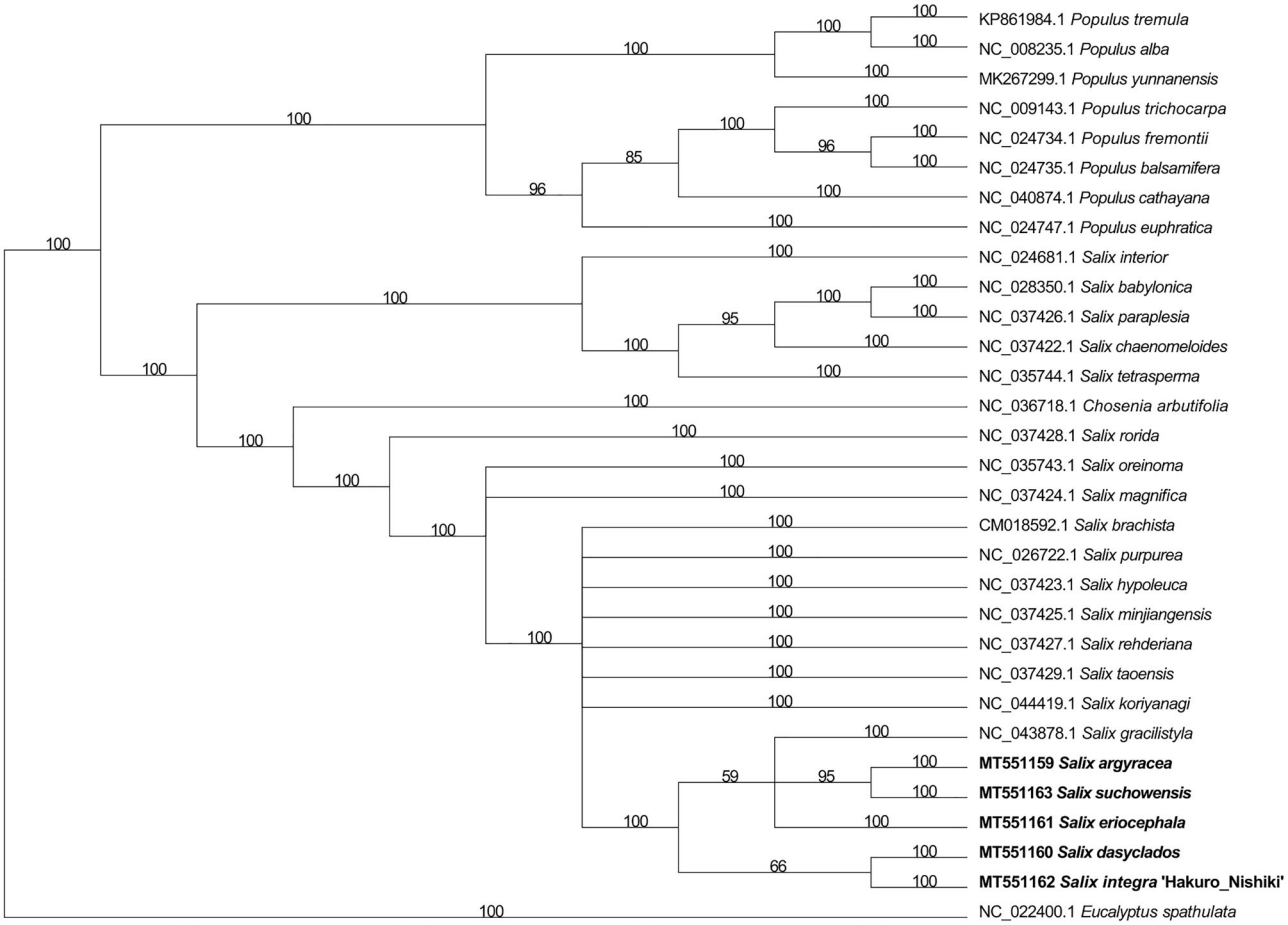
Phylogenic analysis of 21 *Salix* species, *C. arbutifolia*, and 8 *Populus* species based on the complete chloroplast genomes. The maximum likelihood method was based on the auto-model. The bootstrap values are shown next to the branches.

With *Eucalyptus spathulata* as the outgroup, the phylogenetic tree of 21 *Salix* (5 sequenced and 16 published), 1 *Chosenia arbutifolia*, and 8 *Populus* complete cp genomes were constructed using MAFFT (auto mode) (Figure 2). *Salix* formed one robust monophyletic clade. The 21 species within *Salix* were clustered into two subclades. Of the 5 newly sequenced species in this study, *S. argyracea, S. suchowensis,* and *S. eriocephala* were in a clade (together with *S. gracilistyla*)*. Salix dasyclados* was clustered with *S. integra* ‘Hakuro Nishiki’ in a clade. Based on the phylogenetic relationships inferred from the cp genomes, the genus *Salix* in China can be divided into two major groups.

## Discussion

Five *Salix* species were sequenced, and the complete cp genomes of 16 previously published *Salix* species and that of *C. arbutifolia* were annotated. The cp genome size of the five *Salix* species was ∼155 kb and similar to that of the other 17 previously published species (154–156 kb). The GC content of the IR region was high, similar to the previously reported cp genomes of plants (Huang et al. 2017). The results revealed that the structure and synteny of the 21 *Salix* species and *C. arbutifolia* were highly conserved.

Positively selected genes are vital for pinpointing specific targets in adaptive evolution processes, such as environmental, geographical, and host response (Wang et al. 2017). In a photosynthetic organism, loss of activity of *atpF* could impair respiratory activity and affect morphology (Lapaille et al. 2010). The *psaI* encoding photosystem I reaction center subunit VIII indicated that the selection was associated with photosynthesis change in the process of evolution. The *ndhF* exhibited a positive selection effect for its involvement in adapting to hot and dry climates (Carbonell-Caballero et al. 2015; Caspermeyer 2015). These positive selection genes are central to evolutionary patterns and might have driven the successful adaptation of the *Salix* genus.

The taxonomy and systematic phylogeny of the genus *Salix* has been obscure. *Chosenia arbutifolia* was within the clade comprising *Salix* species (Figure 2), which is consistent with previous reports (Chen 2008). In the ‘Flora of China’ (Wu and Raven 1999), *S. dasyclados* and *S. integra* ‘Hakuro Nishiki’ are assigned to the same section as *S. suchowensis* and *S. koriyanagi* are. However, the cp genome data partially support the relationship with traditional taxonomic concepts. The *rps7* gene encodes the ribosome S7 protein, also known as ribosomal protein S7 (*uS7*), which is crucial for the assembly and stability of the ribosome. The *rps7* shows the most variable region among the 21 genomes, indicating that it could be the molecular marker for species identification. Therefore, it is clear that the identification of cp genomes could provide valuable molecular resources for studying the taxonomy and phylogeny of *Salix*. This study provides us with valuable resources, which can be further applied for phylogenetic and evolutionary studies in *Salix*.

## Data Availability

The genome sequence data that support the findings of this study are openly available in GenBank of NCBI at (https://www.ncbi.nlm.nih.gov/) under the accession numbers MT551159 (*S. argyracea*), MT551160 (*S. dasyclados*), MT551161 (*S. eriocephala*), MT551162 (*S. integra* ‘Hakuro Nishiki’), and MT551163 (*S. suchowensis*). The associated BioProject, SRA numbers are PRJNA694772, SRR13528208, SRR13528206, SRR13528205, and SRR13528204, and the Bio-Sample numbers are SAMN17574047, SAMN17574048, SAMN17574049, SAMN17574050, and SAMN17574051, respectively.
